# An Atypical Vascular Complication: Splenic Vein Varix in a Pancreatic Pseudocyst

**DOI:** 10.7759/cureus.90075

**Published:** 2025-08-14

**Authors:** Shivani Iyyappan, Jensy Jaison, Harshavardhan Mahalingam

**Affiliations:** 1 Department of Radiology, Sri Ramachandra Institute of Higher Education and Research, Chennai, IND

**Keywords:** pancreatic pseudocyst, pancreatitis complications, splenic vein, splenic vein varix, vascular anomaly

## Abstract

This report describes the case of a 62-year-old male with a history of idiopathic acute pancreatitis and pseudocyst. Contrast-enhanced computed tomography (CECT) showed a thick-walled pseudocyst arising from the tail of the pancreas. Notably, a hypodense lesion with homogenous enhancement was identified within the posterosuperior aspect of the pseudocyst, communicating with the splenic vein, exhibiting imaging characteristics of a splenic vein varix. There was no sign of active bleeding or splenic vein thrombosis. The patient was managed conservatively, with continued drainage of the pseudocyst, and is on clinical follow-up. This case illustrates the detection of a vascular lesion within a pseudocyst using imaging, which aids in the planning of the patient’s management.

## Introduction

Pancreatic pseudocysts are common sequelae of acute and chronic pancreatitis that develop around four weeks, representing contained peripancreatic fluid collections. It has been referred to by the term *pseudocyst *since the fluid is enveloped by fibrotic granulation tissue and lacks an epithelial lining. While many evolve toward spontaneous resolution, complications such as infection, gastric outlet or biliary obstruction, and hemorrhage can occur, necessitating intervention [1]. Vascular complications in pancreatitis include both venous and arterial events, most notably splenic vein thrombosis (SVT) and arterial pseudoaneurysm. Venous complications, especially SVT, are more common than arterial ones, with a pooled prevalence of venous thrombosis in chronic pancreatitis of approximately 11.6% [2]. Hemorrhage associated with pseudocyst is a particularly feared complication, often resulting from the erosion of an adjacent visceral artery pseudoaneurysm, most commonly the splenic or gastroduodenal artery, into the cyst cavity, leading to intracystic bleeding [3].

While arterial pseudoaneurysms are well-documented in this context, venous varices are significantly rarer, with splenic vein varices being exceptional findings. A venous varix is an abnormal dilatation of a vein. The development of a splenic vein varix within the cavity of a pre-existing pancreatic pseudocyst represents a highly unusual and potentially precarious clinical scenario. While venous pressures are lower than arterial pressures, the weakened wall of the vein contained within the inflammatory environment of a pseudocyst is inherently unstable. The rupture could lead to rapid intracystic bleeding, potentially followed by rupture of the pseudocyst itself into the peritoneum or adjacent structures, resulting in life-threatening hemodynamic compromise [4,5]. Such a finding poses diagnostic challenges in imaging and carries a substantial risk of rupture and life-threatening hemorrhage, distinct from the more typical uncomplicated pseudocyst evolution.

## Case presentation

**Figure 1:**
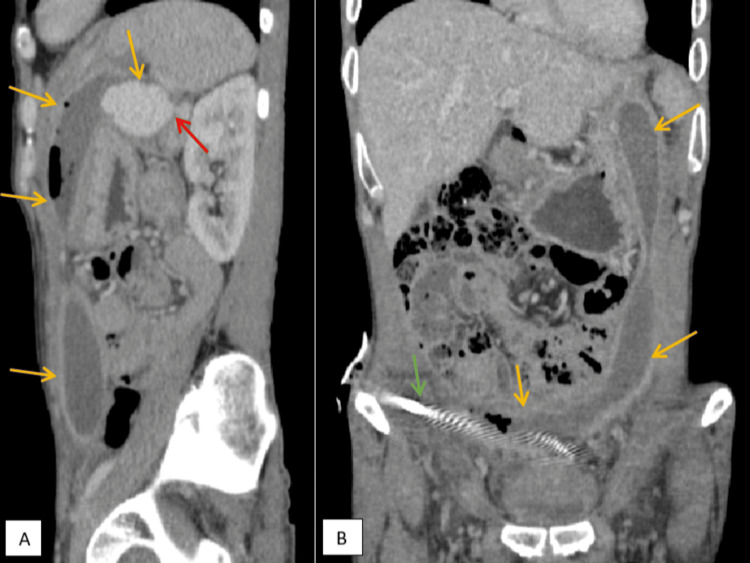
Sagittal (A) and coronal (B) reformatted images from the venous phase of a contrast-enhanced CT abdomen in a 62-year-old male show interconnected, walled-off fluid collections along the left paracolic gutter and pelvis (yellow arrows). The pigtail is seen within the pelvic collection (green arrows). A well-defined lesion is seen within the collection arising from the tail of the pancreas, whose contrast enhancement mirrors that of the splenic vein (red arrow). The lesion is seen arising from the splenic vein, protruding into the pancreatic pseudocyst.

A 62-year-old male with anemia and malnutrition presented with leakage of serous fluid from the site of a previously placed percutaneous pigtail drain for two days. He was a known case of a large pancreatic pseudocyst, a complication of idiopathic acute pancreatitis, that had extended from the tail of the pancreas down into the pelvis. His prior management included endoscopic retrograde cholangiopancreatography (ERCP) with pancreatic duct stenting and percutaneous pigtail catheter drainage of the pelvic component of the collection. He had no history of fever, vomiting, or an increase in the size of his abdomen. His body mass index was 18 kg/m^2^, and his vitals were stable. His abdomen was soft on palpation, and the dressing securing the pigtail was soaked with fluids. His laboratory parameters, such as serum amylase, lipase, and total leukocyte counts, were normal. The hemoglobin was 10.2 g/dL. The initial contrast-enhanced computed tomography (CECT) abdomen of the patient before the pigtail insertion showed a pancreatic pseudocyst occupying the whole abdomen, having around 6,000 mL of fluid. Ultrasonography (USG)-guided pigtail insertion successfully reduced the volume of the collection within three months. A CECT abdomen done after three months at the time of presenting complaints revealed complex interconnected fluid collections amounting to around 300 mL (Figure 1).

**Figure 2:**
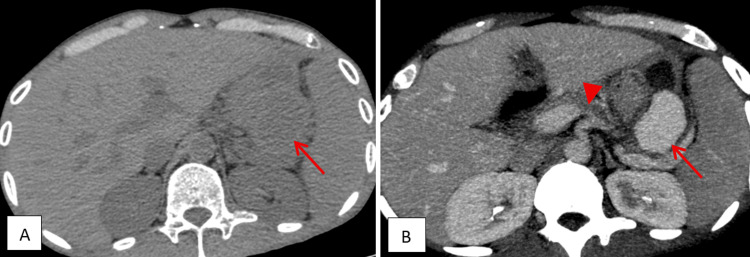
Axial sections of the non-enhanced (A) and venous phase (B) contrast-enhanced CT abdomen show a splenic vein varix within the pancreatic pseudocyst, arising from the splenic vein (red arrow). Narrowing of the splenic vein is seen posterior to the body of the pancreas (red arrowhead).

**Figure 3:**
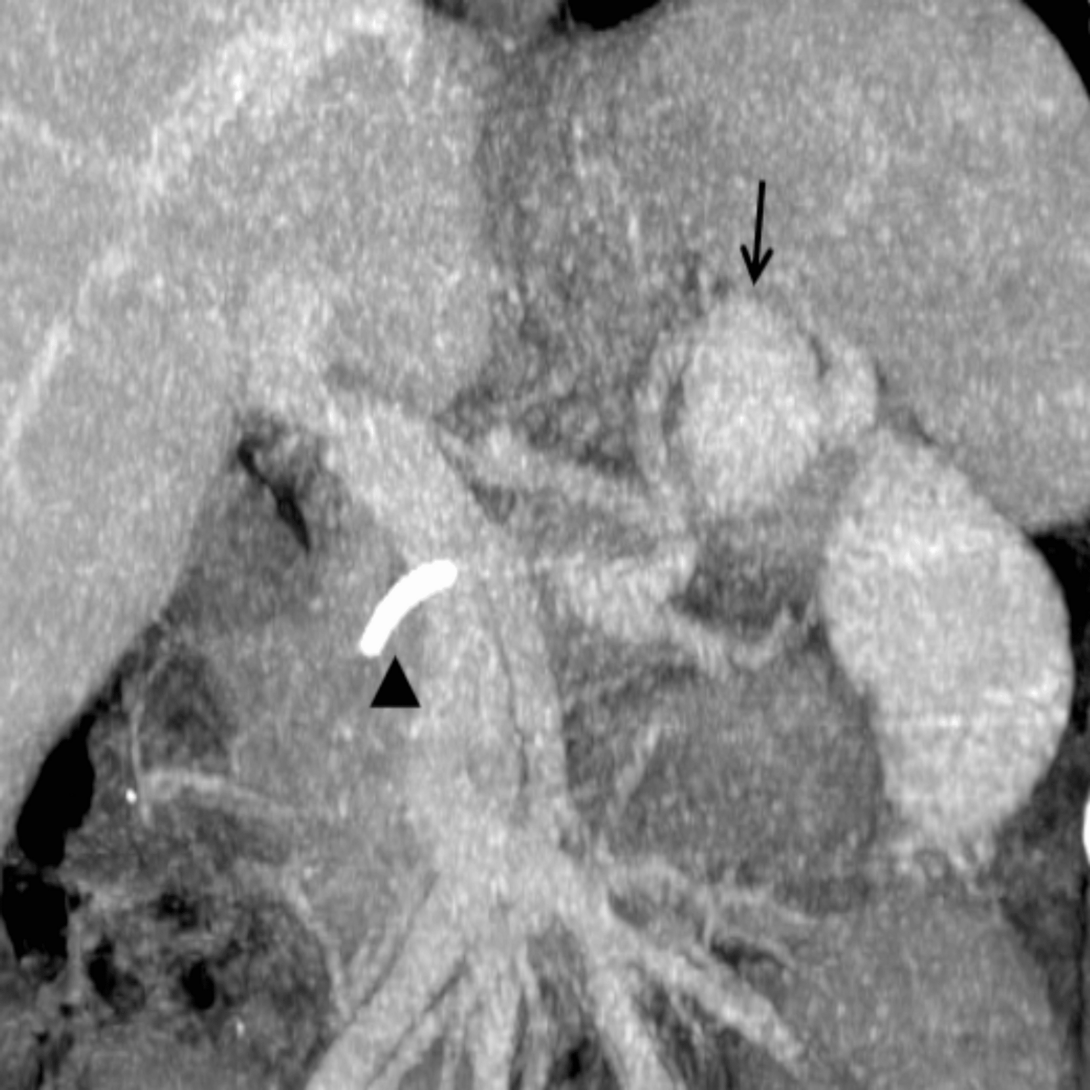
Coronal reconstructed maximum intensity projection (MIP) image from the venous phase of a contrast-enhanced CT abdomen shows the splenic vein varix (arrow). A stent is visible within the main pancreatic duct (arrowhead).

The thick-walled collection in the lesser sac, arising from the tail of the pancreas, contained a distinct hypodense lesion measuring 4.0 × 3.0 × 2.5 cm (Figure 2A). This intracystic lesion demonstrated homogenous enhancement with a mean attenuation of +114 HU, which mirrored the density of the adjacent splenic vein (mean attenuation of +120 HU) on contrast-enhanced phases, particularly the portal venous phase (Figures 1A, 2B, 3). A small communication was identified between this lesion and the splenic vein (Figures 1A, 2B). These findings were interpreted as a splenic vein varix located within the pancreatic pseudocyst, likely secondary to erosion of the splenic vein wall due to mass effect or inflammation from the adjacent pseudocyst. There was no radiological evidence of active contrast extravasation at the time of the scan. The CECT also demonstrated narrowing of the splenic vein caliber posterior to the body of the pancreas (Figure 2B); however, there was no thrombosis of the splenic vein.

The pigtail was within the collection in the pelvis. There was a significant reduction in the size of the collection as compared to the initial time of presentation. The patient was managed conservatively with continued drainage of the pseudocyst through the pigtail. A wait-and-watch approach was undertaken, as the patient was elderly, the pseudocyst had significantly decreased in size following pigtail drainage, and there were no new complaints suggestive of the need for emergency intervention. The patient was on clinical follow-up, where he was advised to note down daily output from the pigtail. He was advised to watch out and immediately seek medical attention in case of a sudden increase in abdominal girth, abdominal pain, vomiting, or altered sensorium. Ultrasound follow-up of the collection size was advised on a monthly basis or when there is no output from the pigtail. After one month, the pigtail output was negligible for two days. On ultrasound screening, the collection had resolved. Hence the pigtail was removed. In cases of asymptomatic splenic vein varix, follow-up every six months, then annually, ideally with triphasic CECT of the abdomen, was advised.

## Discussion

The presence of an intracystic splenic vein varix carries significant clinical implications. First, it poses a diagnostic challenge. On imaging, particularly CECT, which is the mainstay for evaluating pancreatic pseudocysts, differentiating intracystic varices from other complex cystic features like debris, clots, or septations requires scrutiny of contrast enhancement patterns. It is important to trace the vessel of origin of this vascular anomaly, particularly in the presence of complications seen in pancreatitis, like splenic vein thrombosis and splenic artery pseudoaneurysm. As demonstrated in our case, the lesion's homogenous enhancement mirroring the splenic vein, coupled with the identifiable communication with the vein, was a key feature leading to the diagnosis [6]. Doppler ultrasound or angiography could potentially offer further characterization, but may not always be necessary if CECT findings are clear. The closest differential for this would be a splenic artery pseudoaneurysm, which would arise from the splenic artery and enhance with attenuation values similar to the splenic artery. Other least likely differentials for the intracystic lesions could be organized debris or clot, which would have variable attenuation and would not enhance in contrast studies. Second, and most critically, an intracystic splenic vein varix represents a high-risk situation for catastrophic hemorrhage [4].

The management of splenic vein varices is not well standardized due to their rarity. Given the risk of rupture, the management of intracystic splenic vein varix often involves prompt intervention aimed at excluding the lesion from circulation. Trans-arterial embolization is frequently the first line for arterial pseudoaneurysms, while options for venous varices, like splenic vein varix, might include endovascular approaches or definitive surgery, often involving distal pancreatectomy with splenectomy. Management strategies for splenic vein varix associated with pancreatitis range from conservative observation, rarely appropriate for venous varices, especially intracystic [7], to endovascular techniques like stent placement [8-10], to surgical resection [5].

Interestingly, in the case presented, a conservative approach was adopted regarding the splenic vein varix. The immediate management focused on securing the existing pelvic drain and continuing drainage of the extensive pseudocyst. This decision diverges somewhat from the typical urgency associated with managing intracystic splenic vein varix due to its recognized bleeding potential. The rationale for this conservative approach towards the splenic vein varix involved multiple factors. These included the patient's clinical stability at presentation, the absence of active contrast extravasation on the CECT scan, and potentially high perceived risks associated with immediate endovascular or surgical intervention in the setting of good clinical response to minimally invasive pigtail drainage of the large pseudocyst. A strategic decision to attempt pseudocyst decompression via drainage, potentially reducing pressure on the splenic vein varix, was taken, keeping in mind the patient's comorbidities.

However, opting for conservative management of an intracystic splenic vein varix necessitates extremely close clinical and potentially radiological surveillance. As in this case, clinical follow-up included daily monitoring of pigtail output, and the patient was advised to watch for sudden increases in abdominal girth, abdominal pain, vomiting, or altered sensorium. Ultrasound screening was mainly focused on pseudocyst drainage.

## Conclusions

This case illuminates the rare occurrence of a highly uncommon vascular complication, an intracystic splenic vein varix within a pancreatic pseudocyst. A hypodense lesion with homogeneous enhancement mirroring the splenic vein, together with an identifiable communication with the vein, is a key feature supporting the diagnosis of splenic vein varix. The identification of this represents a critical finding, as its presence increases the risk of catastrophic hemorrhage that demands a very high clinical suspicion to ensure timely recognition and intervention. Accurate differentiation from arterial pseudoaneurysms, debris, or thrombus relies on imaging modalities like CECT and Doppler ultrasound, focusing on enhancement patterns and vascular continuity. Advanced imaging, notably CECT, emerges as an indispensable tool in delineating the anatomical and vascular characteristics of a varix, thereby enabling strategic clinical decisions. In this instance, a conservative strategy was employed in view of the patient’s clinical status. However, such an approach mandates meticulous clinical and radiological follow-up, given the persistent threat of varix rupture and subsequent life-threatening bleeding.

In conclusion, this case exemplifies how even commonplace clinical entities may harbor rare complications. It reinforces the necessity of careful diagnosis, interdisciplinary collaboration, and individualized therapeutic planning in managing complex pancreatic pseudocysts, thereby optimizing patient outcomes in the face of unexpected clinical challenges.
